# Isolated Volar Radioulnar Joint Dislocation With Associated Ulnar Styloid Fracture

**DOI:** 10.7759/cureus.61977

**Published:** 2024-06-08

**Authors:** Arman Israelyan, James Chiang, Kassandra C Cooper, Valerie L Lew, Gary C Murphey, Edward Durant

**Affiliations:** 1 Emergency Department, Kaiser Permanente Modesto Medical Center, Modesto, USA

**Keywords:** ulnar nerve block, ulnar styloid fracture, ultrasound-guided, volar druj dislocation, distal radioulnar joint dislocation

## Abstract

Isolated volar dislocation of the distal radioulnar joint is a rare occurrence and is commonly missed. The mechanism of injury typically involves hypersupination. True lateral radiographs are difficult to obtain as patients are usually limited with wrist pronation and supination, resulting in a high miss rate. We describe a 32-year-old male who presented to the emergency department (ED) with pain and swelling of the posteromedial aspect of the right wrist after punching a wall one hour prior to presentation. Examination revealed soft tissue tenderness and mild edema at the right distal ulna with an associated deformity, best visualized at the volar aspect of the right wrist. Active range of motion was limited with right wrist flexion and extension, secondary to pain and edema. Right wrist supination and pronation strength and range of motion were limited due to the patient’s tenderness on examination. Peripheral nerve function and vascular examination were normal. Initial radiographs of the right hand, wrist, and forearm did not reveal a fracture or dislocation. A musculoskeletal computed tomography (CT) scan of the right hand and wrist revealed an avulsion fracture of the ulnar styloid with volar displacement of the ulna. Analgesia was achieved with an ultrasound-guided ulnar nerve block, and the right wrist was successfully reduced. This report highlights the difficulty in obtaining a diagnosis of an isolated volar dislocation of the distal radioulnar joint. We recommend obtaining a musculoskeletal CT scan in the setting of an inconclusive radiograph and incongruent physical examination. Analgesia can also be achieved with an ulnar nerve block under ultrasound guidance.

## Introduction

Isolated volar dislocation of the distal radioulnar joint (DRUJ) is a rare occurrence and is commonly missed [1]. According to a systemic review conducted in 2022 by O’Malley et al., this injury represents nearly 0.02% of all orthopedic injuries and is diagnosed later in 36% of cases, although some literature reviews have quoted a figure of 50% [2]. The mechanism of injury typically involves hypersupination [2,3]. True lateral radiographs are difficult to obtain, as patients are usually limited with wrist pronation and supination, resulting in a high miss rate [2]. In this case report, we highlight a 32-year-old male who presented to the emergency department (ED) with a traumatic volar DRUJ dislocation with an associated ulnar styloid fracture.

## Case presentation

A 32-year-old male with a history of hypertension, mild persistent asthma, and obstructive sleep apnea presented to the ED with pain and swelling of the posteromedial aspect of the right wrist after punching a wall one hour prior to presentation. He is right hand dominant and denies any previous history of fractures or dislocation of the extremities. The patient reports some limitations with wrist flexion, extension, abduction, and adduction but denies any weakness or numbness in the involved extremity. He also reports no limitations with active range of motion at the right elbow and shoulder.

Vital signs revealed an initial blood pressure of 149/113 mmHg, pulse of 63 beats per minute, temperature of 97.2°F (36.2°C), respiratory rate of 20, saturation of peripheral oxygen level of 98% on room air, and a weight of 105.5 kg with a body mass index of 33.37 kg/m^2^.

Musculoskeletal examination revealed soft tissue tenderness and mild edema at the right distal ulna with associated deformity, best visualized at the volar aspect of the right wrist. Active range of motion was limited with right wrist flexion and extension, secondary to pain and edema. Radial pulse was 2+. The patient demonstrated adequate hand grip strength. He demonstrated normal median nerve functioning with an “okay” sign. Interossei muscle testing was within normal limits, demonstrating normal ulnar nerve functioning. Patient also demonstrated normal digit and thumb extension against resistance, indicating normal radial nerve functioning. There were no sensory deficits at the right upper extremity. Both active and passive range of motion at the right elbow and shoulder revealed no abnormalities. The patient demonstrated normal strength with right elbow extension and flexion. Right wrist supination and pronation strength and range of motion were limited due to patient’s tenderness on exam. 

**Figure 1:**
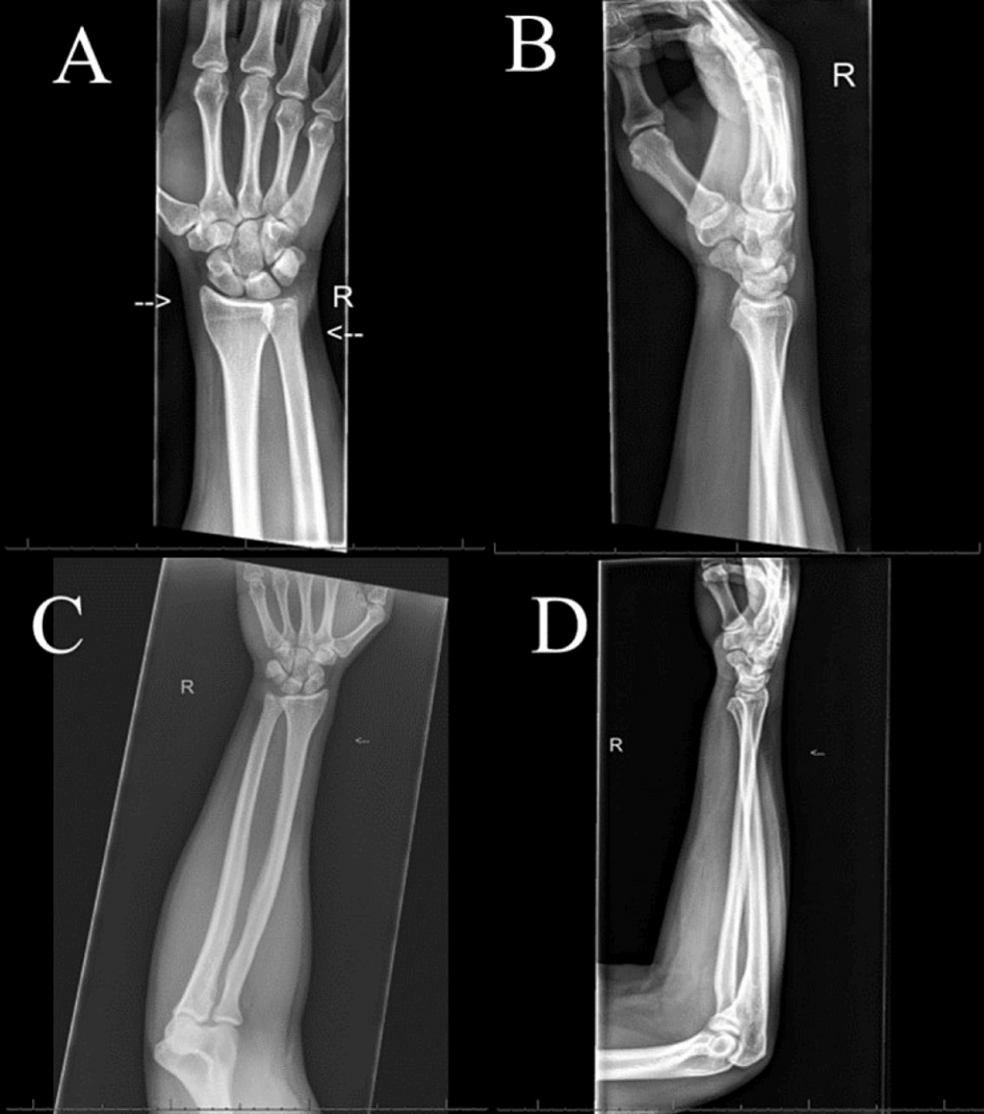
Anterior/posterior and lateral radiographs of the right wrist and forearm. (A and B) Anterior/posterior and lateral radiograph views of the right wrist, respectively, prior to reduction. (C and D) Anterior/posterior and lateral radiograph views of the right forearm, respectively, prior to reduction. These images were initially interpreted as normal but later addended “on the frontal view, there is overlapping of the DRUJ on AP view but looks normal on lateral view, which may be due to suboptimal positioning or DRUJ is unstable and partially subluxed/(dislocated) depending on positioning.”

**Figure 2:**
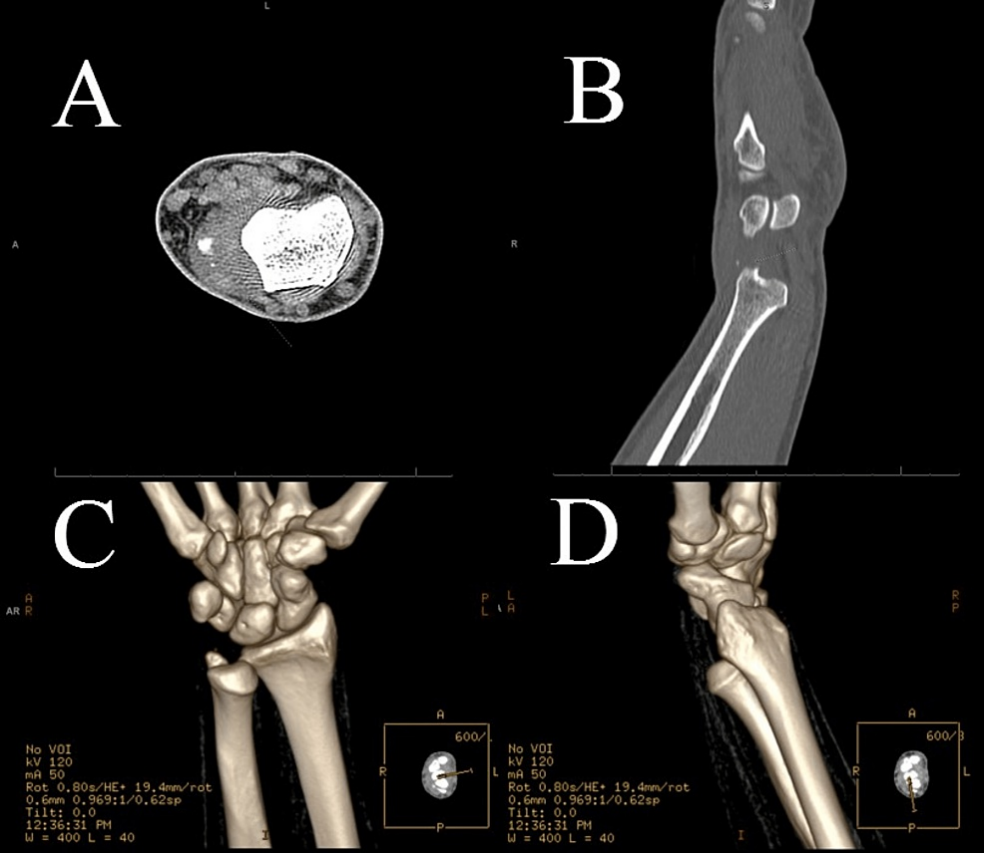
Musculoskeletal CT axial, sagittal, and three-dimensional images of the right hand and wrist. (A and B) Axial and sagittal views via musculoskeletal CT of the right hand and wrist. (C and D) Three-dimensional images via musculoskeletal CT of the right hand and wrist. These images were initially interpreted as “a small calcific density along the volar aspect of the ulna styloid which may represent avulsion fracture fragment.” The addendum later notes a “volar dislocation of the ulna with punctate avulsion fracture fragment along the dorsal aspect at the level of the ulnar styloid.” CT: computed tomography.

Initial radiographs of the right hand, wrist and forearm revealed no acute fracture or dislocation per the radiologist (Figure 1A-D). However, the ED physician noted a slight increase in overlap of the DRUJ and has a high clinical suspicion of dislocation or fracture. Therefore, a musculoskeletal computed tomography (CT) scan of the right hand and wrist was obtained and revealed a dislocation of the DRUJ with an avulsion fracture of the ulnar styloid with soft tissue swelling and edema surrounding the site (Figure 2A-D).

The patient was administered one tablet of 5-325 mg oxycodone-acetaminophen and 30 mg of intramuscular ketorolac with near relief of pain.

**Figure 3:**
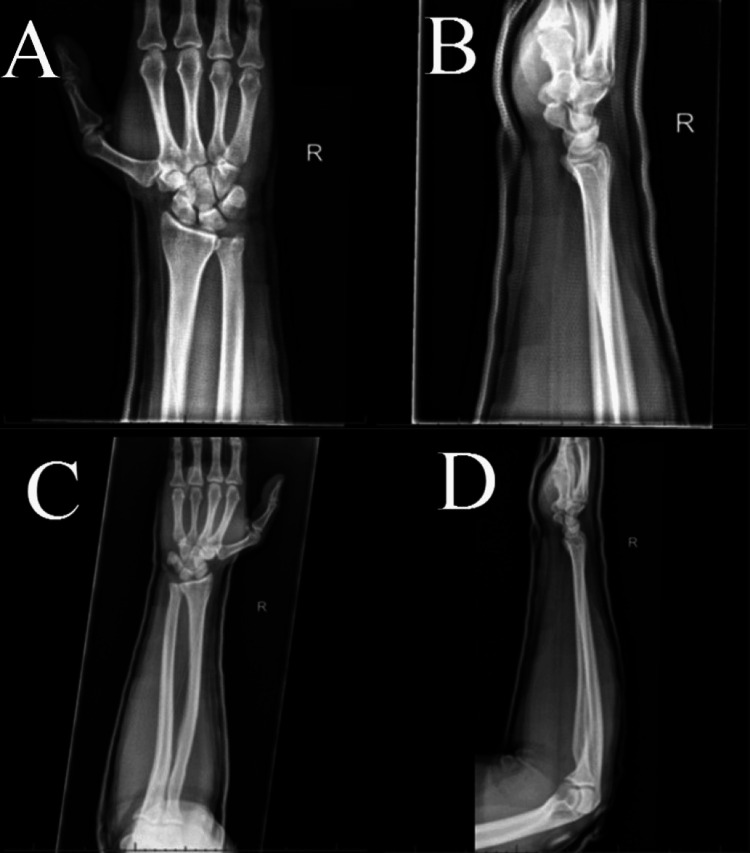
Anterior/posterior and lateral radiographs of the right wrist and forearm after reduction. (A and B) Anterior/posterior and lateral radiograph views of the right wrist, respectively, after reduction. (C and D) Anterior/posterior and lateral radiograph views of the right forearm, respectively, after reduction. These images revealed satisfactory alignment per the radiologist.

Written consent was obtained for a right peripheral ulnar nerve block and a right wrist reduction. An ulnar nerve block was successfully performed 2 cm distal to the ulnar groove under ultrasound guidance with 0.5% bupivacaine-epinephrine 1:200,000. The right wrist was reduced using traction-countertraction, and an anterior-posterior force was directed towards the ulnar head with slight wrist extension. The neurovascular examination remained normal after the reduction. He was placed in a sugar tong splint. A post-reduction radiograph revealed improved DRUJ and ulnar shaft alignment (Figure 3A-D). The patient was discharged home in stable condition with a referral to Orthopedics.

**Figure 4:**
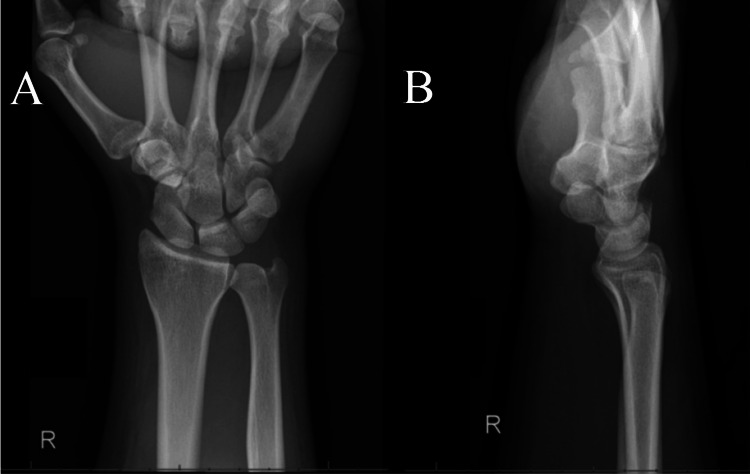
Anterior/posterior radiographs of the right wrist five days after reduction. (A and B) Anterior/posterior and lateral radiograph views of the right wrist, respectively, five days after reduction. These images were stable from prior and revealed no occurrence of dislocation.

Radiographs obtained five days later in the outpatient setting remained unchanged (Figure 4A, B). Active range of motion of the wrist in all planes is intact with no signs of DRUJ instability.

## Discussion

This case highlights the difficulty of diagnosing an acute isolated dislocation of the DRUJ. It also emphasizes the rarity of a DRUJ dislocation with an associated ulnar styloid fracture as these injuries typically involve the distal radius [5]. According to our literature review, there are only two case reports that describe a similar fracture pattern [5,6]. 

Radiographic evaluation of acute DRUJ pathology begins with posteroanterior and lateral films of the wrist. Relevant findings include widening of the DRUJ space and a radioulnar distance greater than 6 mm, respectively [7]. CT scans should be utilized in the appropriate clinical context, particularly if the quality of the lateral radiograph is lacking or if the patient is unable to rotate the forearm [2]. According to Yang et al., a true lateral radiograph is defined as the “overlaying of the palmar cortex of the pisiform and the central third of the interval between the palmar cortices of the distal scaphoid pole and capitate head” [4]. This approach serves as a diagnostically reproducible standard but is not often adhered to in the setting of limited forearm rotation. In this case, a musculoskeletal CT scan confirmed the clinical suspicion of a fracture or dislocation and aided in the diagnosis.

Historically, treatment was achieved by closed reduction under sedation or anesthesia in the emergency setting [1]. Closed reduction of a volar dislocation is often difficult to achieve as the clinician must overcome the pull of the pronator quadratus [5]. In a case series described by O’Malley et al., a widely adopted technique includes a combination of traction, dorsal pressure on the ulnar head, and forearm pronation. One can also utilize the "Boyer method" to aid in reduction, introduced in 1912, where fingers are placed directly in between the radius and ulna to distract the bones first. If the DRUJ is stable after reduction, immobilization is recommended for four to six weeks in an above elbow sugar-tong cast [2,8]. Surgery is indicated if the dislocation persists or if there are signs of DRUJ instability [1]. In our report, complete anesthesia was achieved with an ultrasound-guided ulnar nerve block. These procedures have been proven to be safe and effective in the management of upper and lower limb emergencies if performed by emergency physicians with adequate training [9,10]. Local anesthesia under ultrasound guidance proved to be feasible, safe, cost-effective, and provided comfort and analgesia through discharge. 

## Conclusions

We describe a 32-year-old male with an isolated volar DRUJ dislocation with an associated ulnar styloid fracture. This report highlights the difficulty in obtaining this diagnosis. We recommend obtaining a musculoskeletal CT scan in the setting of an inconclusive radiograph and incongruent physical examination. Analgesia can also be achieved with an ulnar nerve block under ultrasound guidance.
